# The Illinois Veterinary Medical Association

**Published:** 1892-01

**Authors:** Matthew Wilson

**Affiliations:** Recording Secretary; Mendota, Ill


### SOCIETY PROCEEDINGS.

*The Illinois Veterinary Medical Association.*—The Illinois State Veterinary Medical Association held its regular annual meeting, November 17th and 18th at the Sherman House, Chicago, Ill., being presided over by President S. S. Baker of Chicago.

On account of unavoidable absence of the Secretary and Treasurer, the President appointed Dr. M. Wilson Secretary *pro tem* and Dr. N. I. Stringer, Treasurer *pro tem.*

The roll being called the following members responded: Drs. A. H. Baker, S. S. Baker, G. Z. Barnes, H. E. Delavergne, Thomas Hope, J. A. Judson, O. J. Lanigan, W. J. Martin, J. W. Parkinson, C. A. Pierce, H. G. Pyle, J. F. Ryan, C. E. Sayre, J. S. Spangler, R. W. Story, R. J. Withers, S. V. Ramsey, A. G. Alverson, D. McIntosh, N. I. Stringer, M. Wilson, R. G. Walker, F. S. Schoenleber, T. J. Gunning.

The minutes of the previous meeting were read and approved, and correspondence from Drs. J. T. Nattress, J. F. Pease, Paul Paquin and S H. Kingery were read regretting their absence.

President S. S. Baker then gave his annual address as follows:

"Gentlemen;—This Association has entered on the ninth year of its existence. We have held meetings during these nine years, when we have had barely a working quorum, and one meeting in particular, I remember, held at Joliet in 1885, where there were but six members present, not enough for a quorum, but we did more business at that meeting than was ever done before or since; we revised Constitution and By-laws, etc., etc., only to have our work thrown out at the next meeting as illegal, but of late we have had no such trouble; we have gained in the number of our members; the zeal with which they attend has grown with the increased membership. The meetings of this Association are not merely for the purpose of mutual admiration, but for a specific object; viz.: the cultivation of fraternal feeling among Veterinary practitioners, the elevation of the Veterinary science to an equal rank with other scientific branches of medicine, the mutual improvement of its members, by the exchange of ideas, the presentation of such cases of diseases together with their treatment, as may be worthy of note, and to provide ways and means to secure legslation to regulate the practice of veterinary medicine and surgery in this state. The importance of such legislation is not so much for the protection of the qualified practitioner, as for the protection of the Agriculturist against the ignorant empirical practitioner.

"Because we failed once in securing legislation, is no reason why we should give up in despair. The medical profession strove for ten years before they got a bill passed to regulate the practice of medicine, therefore, I would urge upon you the appointment of a committee for that purpose, and each of you get your representative interested in the work, and keep at it till we get the desired legislation. The advancement in the veterinary profession in the last few years has been great. We are now recognized by the Federal Government and by most all state governments. We have members of our profession who hold federal appointments not only at Washington, but at all the important seaports, and also in England; some of the states have veterinarians at the slaughter houses, to inspect the cattle before they are killed, and the meat afterwards. A short time ago the Federal Government gave each state that had a university $15,000.00 for the purpose of making original investigations in the diseases of animals. Nebraska established a labratory, and engaged a man for that purpose, the result of which you all are well aware. What has Illinois done? Nothing. The $15,000.00 was given the university at Champaign, and we, as veterinarians have a right to demand that something be done with that money. The success or our meetings is entirely with the members themselves, in the interest they show in attendance, and in the participation of the active duties of the Association. I sincerely hope that with our revised by-laws and new code of ethics, new life will be instilled in the members, that more interest will be taken in the workings of the Association, and I also hope that each member will consider himself a committee of one to secure new additions to our membership, thus increasing our numbers till we have all the qualified practitioners in the state within the folds of the Association.

"I would also urge upon you the importance of becoming members of the United States Veterinary Medical Association, thereby assisting to make that body a truly national one. The State Associations can only hope to have a local influence, but the U. S. V. M. A. is bound to wield an influence that will be felt throughout the length and breadth of the land. I think it only a question of time, when it will be a requisite condition to qualify a man to become a member of that body, that he be a member in good standing in a state association. The benefits to be derived from these meetings are many, a man gets an opportunity to gain new ideas, it takes him out of the old rut he has been running in, gives him a chance to get the dust blown off his best suit of clothes, he sees new faces, puts more money into circulation, and if he is not troubled with big head, (not lump jaw) he will learn something, he enjoys himself and goes home with fresh vigor to again take up the task of life.

"I tell you, gentlemen, in these days of Microbes, it is not safe for a man to stay away from a meeting, when he may learn something about that which is at the bottom of all diseases, for everything is germs now.

"I beg to thank you, gentlemen, most sincerely for the honor you conferred on me, in electing me president of this association for the past year. I wish also to thank the different members of the programme committee for their great assistance in aiding me to place before you a programme which I feel will be both interesting and instructive."

The Secretary being absent no report was given.

A report from Dr. J. T. Nattress showed a cash balance on hand of $34.35

There being no committees to report, the President called on Dr. M. Wilson to read his paper on "Influenza." After a brief discussion chiefly regarding the treatment, the discussion was closed on motion. *Vide fol. 21.*

Article 4, Sec. 1, of the constitution, which was revised at last meeting, was adopted on vote of yeas and nays. The revised by-laws, except article 9, were also adopted. On motion article 9 of the by-laws was laid over until next day.

After a ten-minute recess the President called on Dr. G. Z. Barnes, of Pekin, Ill., to read a paper on "Tetanus."

Dr. Barnes defined tetanus as an extra-organismal infectious disease, with spasmodic contraction of the voluntary muscles. He believes acute tetanus to come from traumatic causes. He then described the symptoms in the acute and the subacute forms. He referred to the discovery of Ta Kitasata, who in Koch's labratory isolated the pure germs of the disease and established their identity with those taken from a wound in a horse's foot, and he reviewed the experiments of Theo. Kitt, of Munich, which prove the infectiousness of the disease ; the discovery of Dr. Bassano of the germs in the soil from twenty-seven different countries ; and the observations of Sormani on the action of the digestive tract, which show that ; 1st, the flesh of animals that have died of tetanus can be eaten with impunity ; 2nd, the microbes of tetanus can pass through the alimentary canal of herbivorous and carnivorous animals without causing any symptoms ; 3d, the digestive secretions of these animals neither kill nor alter in any way the bacillus of tetanus ; 4th, an animal can introduce into its stomach with impunity a dose of tetanic virus two thousand times greater than that which is sufficient to kill if injected subcutaneously. Drs. Behring and Kitasita have made positive advances towards the production of immunity against tetanus, and arresting the disease when once established.

We believe we are safe in arriving at the following conclusions :

First, tetanus is a specific infectous disease. Second, out side of the diseased organism the specific germ may develop in the soil. Third, in addition it may exist at different points, on the coats of horses or other animals, in the dust of hay, surgeons instruments, etc. Fourth, these different situations (soil, dust, horse, etc.) constitute a closed cycle from any element of which the bacillus may constantly or indefinitely emigrate, so that it is not possible to designate the point of initial departure. Fifth, the horse plays in this transmission no greater role, than cattle in the propogation of tuberculosis.

As to the treatment of tetanus we have nothing particularly new to offer. Unfortunately the disease is well under way before a veterinarian is called.

Although I generally prescribe gelseminum and cannabis indica, alternating with large doses of Fowler's solution of arsenic, I am firmly of the belief that simple treatment is the much more rational method to employ, rather than the vain hope of finding a specific, to be constantly recurring to antispasmodics and sedatives, which repeated experience has proven to be useless as curative agents.

Many experiments have been made and nitrate of silver has been found to be a powerful agent in the destruction of the bacillus of tetanus, hence its therapeutic value in treating wounds when there is a possibility of tetanus following. Dr. Sormani recommends iodoform as a wound dressing in the disease, claiming that it neutralizes perfectly the poison of tetanus.

After a discussion relating almost entirely to the various forms of treatment, the discussion was closed on motion.

Dr. A. H. Baker was then called upon, and the subject he chose was the post mortem appearance of the cattle disease, which Dr. Caswell, Dr. Williams, and himself investigated in various parts of the state this past summer.

The motion to adjourn until next morning was carried.

Novemder 18, 1891. The meeting being called to order at 10:00 A. M., the president called on Prof. McIntosh to read his paper on "Parturient Apoplexy." *Vide fol. 16.*

After a lengthy discussion regarding the pathological cause and the treatment, the discussion was closed on motion.

Dr. Schoenleber was then called upon to read his paper on Veterinary Agricultural Editorials. After some remarks upon the subject by the members the discussion was closed. A letter was read from Dr. Williams regretting his inability to be present, and tendering his resignation as a member of the association on account of his now being a non-resident of the state. Dr. Williams' resignation being accepted, on motion by Dr. Stringer seconded by Dr. Ramsey, he was made an honorary member. The Secretary was instructed to write a letter to Dr. Williams testifying our sincere regret at his having to resign his active membership, and to thank him for his past labors in behalf of the association.

Article 9 of the by-laws, The Code of Ethics, was read and on motion by Dr. Ramsey, seconded by Dr. Delavergne, was adopted.

The meeting then adjourned until after dinner.

The afternoon session being called to order Dr. Withers was called upon to read his paper on "The Use of Antiseptics."

After some remarks upon the use of antiseptics in the various surgical cases the discussion was closed. Dr. O. J. Lanigan, of Wenona, then gave a case report. *Vide fol. 26.*

Dr. J. A. McDonnell of Chicago read a paper on the Veterinary profession, giving its history from the early period to the present time. He particularly called attention of his hearers to thoroughly recognize the importance of their vocation and thus to gain from the general public a recognition placing them on an equal footing with the exponents of every other branch of medical science.

G. B. C. Klophel, M.D., of Chicago, then read a paper on Tuberculosis. The Doctor commenced by stating that it was the first time that he he had been honored by an invitation from a Veterinary Association, and was pleased to meet such a body of distinguished men as he saw before him.

After a lengthy discourse on the history of the disease he recommended a thorough and systematic inspection of meats intended for use and exportation, and also a thorough inspection of milk by competent veterinarians. A vote of thanks was extended to Drs. McDonnell and Klophel, and both made honorary members as a recognition of their endeavors to benefit the Veterinary profession.

The following gentlemen's names were then proposed for membership: Drs. F. H. Anderson (Ontario, '89), Evanston; Otis Barnett (Chicago, '91), Edwardsville; Walter Allen (Chicago, '90), Dunlap; A. M. Story (Ontario, '91), Buda; F. L. Brown (Chicago, '89) Galesburg. On motion by Dr. Stringer, seconded by Dr. Gunning, they were unanimonsly elected.

The following officers were then elected for the ensuing year: President, Dr. S. S. Baker; Vice-President, Dr. S. V. Ramsey; Secretary, Dr. M. Wilson; Treasurer, Dr. N. I. Stringer; Censors, Drs. Lanigan, Nattress and Walker.

The President appointed the following standing committees:

Committee on Programme, Drs. Pyle, Schoenleber and Story. Committee on Arrangements, Drs. Lanigan and Gunning. Committee on Legislation, Drs. Walker, Nattress and Pease. Committee on Membership, Drs. Stringer, Thomson and Alverson.

It was moved and seconded that a committee consisting of Drs. Pyle, Schoenleber and Parkinson see that the Code of Ethics be complied with by members of the association and report at each meeting. Carried on motion. A proposition, signed by three members, to change Sec. 3, Art. II, of the By-Laws, relating to the number of members necessary to form a quorum, from the present number (10) to five, was laid on the table.

A vote of thanks was tendered the retiring officers. The association adjourned to meet in Peoria at the call of the Committee.

MATTHEW WILSON, M. R. C. V. S,

*Recording Secretary.*

MENDOTA, ILL, December 4th, 1891.

